# Ribociclib Improves Overall Survival in HR+/HER2– Metastatic Breast Cancer Across Common Genomic and Clinical Subtypes

**DOI:** 10.1093/oncolo/oyac010

**Published:** 2022-03-28

**Authors:** Anne Jacobson

## Abstract

In updated biomarker and clinical subgroup analyses from the phase III MONALEESA trials, ribociclib demonstrated consistent improvements in overall survival when added to endocrine therapy in patients with HR-positive/HER2-negative advanced breast cancer.

When added to standard endocrine therapy (ET), cyclin dependent kinase 4/6 (CDK4/6) inhibitors improve outcomes for patients with hormone receptor (HR)-positive, human epidermal growth factor receptor 2 (HER2)-negative advanced and metastatic breast cancer.

Treatment with the CDK4/6 inhibitor ribociclib plus ET significantly improved overall survival (OS) relative to ET alone in the landmark phase III MONALEESA-2, MONALEESA-3, and MONALEESA-7 trials, which represented the spectrum of premenopausal and postmenopausal patients with HR-positive, HER2-negative advanced breast cancer.^1–3^

Understanding how the magnitude of benefit with CDK4/6 inhibitor therapy varies by clinical and genomic subtype may enable oncologists to develop personalized treatment plans. In subgroup analyses from the MONALEESA trials, researchers evaluated OS outcomes by intrinsic tumor subtype and metastatic site.^4,5^

## Ribociclib and Overall Survival by Intrinsic Tumor Subtype

Lisa A. Carey, M.D., of the University of North Carolina at Chapel Hill, presented results from an analysis of overall survival in the MONALEESA-2, -3, and -7 trials by intrinsic tumor subtype.^4^

In the retrospective exploratory analysis, tumor samples from patients in the MONALEESA trials (*n* = 997) underwent gene expression profiling with the Prediction Analysis of Microarray 50 (PAM50) assay. Luminal A was the most common subtype (54.4%), followed by luminal B (27.9%), HER2-enriched (14.7%), and basal-like (3.0%).

Across all patients, ribociclib added to ET significantly improved OS by 25% compared with ET alone (HR, 0.75; *p* .0012). However, results showed a significant interaction between tumor subtype and survival benefit (*p* = .0065) (Table 1). Ribociclib significantly improved OS for patients with luminal A (HR, 0.75; *p* = .021), luminal B (HR, 0.69; *p* = .023), and HER2-enriched tumor subtypes (HR, 0.60; *p* = .018). In contrast, ribociclib did not improve OS relative to placebo in patients with basal-like tumors (HR, 1.89; *p* = .148).

**Table 1.** Overall survival by intrinsic tumor subtype and adjuvant therapy

Tumor subtype	Ribociclib plus ET	ET alone	HR	*p* value
Luminal A (*n* = 542)	68.0 months	54.6 months	.75	.021
Luminal B (*n* = 278)	58.8 months	44.9 months	.69	.023
HER2E (*n* = 147)	40.3 months	29.4 months	.60	.018
Basal like (*n* = 30)	19.4 months	21.2 months	1.89	.148

Abbreviations: ET, endocrine therapy; HER2E, HER2 enriched; HR, hazard ratio.

Intrinsic tumor subtype was prognostic for survival outcomes (*p* < .001) after adjusting for clinical covariates. Patients with the basal-like subtype did not appear to benefit from CDK 4/6 inhibitor therapy, whereas those with HER2-enriched tumors experienced the greatest magnitude of survival benefit.

The phase III HARMONIA trial will evaluate the activity of ribociclib plus ET in patients with HER2-enriched tumors, with the goal of providing further insight on developing personalized treatment plans for patients with HR+/HER2– metastatic breast cancer.

## Ribociclib and Overall Survival by Metastatic Site

The phase III MONALEESA-2 trial evaluated first-line ribociclib plus letrozole, compared with placebo plus letrozole, in 668 postmenopausal women with HR+/HER- advanced breast cancer. In the overall study population, ribociclib significantly improved OS by 24% compared with ET alone (HR, 0.76; *p* = .004).^1^

Joyce O’Shaughnessy, M.D., of Texas Oncology-Baylor University Medical Center, presented findings from an exploratory analysis of OS by metastatic site in the MONALEESA-2 trial.^5^

Results showed a consistent survival benefit in favor of ribociclib plus letrozole relative to letrozole alone, regardless of metastatic site (Table 2). All comparisons favored treatment with ribociclib, though some comparisons did not reach statistical significance.

**Table 2.** Overall survival by metastatic site in HR+/HER2– advanced breast cancer

Metastatic site	Ribociclib plus letrozole	Placebo plus letrozole	HR (95% CI)
Bone-only metastases			
Yes	(*n* = 69)	(*n* = 79)	
Median OS	72.6 months	56.4 months	0.78 (0.50–1.21)
6-year OS	50.2%	33.8%	
No	(*n* = 265)	(*n* = 255)	
Median OS	61.5 months	50.3 months	0.77 (0.51–0.96)
6-year OS	42.6%	31.5%	
Liver metastases			
Yes	(*n* = 59)	(*n* = 72)	
Median OS	37.7 months	38.1 months	0.81 (0.54–1.24)
6-year OS	31.0%	18.9%	
No	(*n* = 275)	(*n* = 262)	
Median OS	68.0 months	56.9 months	0.77 (0.62–0.97)
6-year OS	46.8%	35.7%	
Liver or lung metastases			
Yes	(*n* = 182)	(*n* = 190)	
Median OS	55.5 months	51.4 months	0.81 (0.62–1.05)
6-year OS	31.0%	18.9%	
No	(*n* = 152)	(*n* = 144)	
Median OS	70.5 months	52.4 months	0.71 (0.53–0.96)
6-year OS	48.6%	33.2%	

Abbreviations: CI, confidence interval; HR, hazard ratio; OS, overall survival.

Results also favored treatment with ribociclib regardless of the number of metastatic sites. Compared with letrozole alone, ribociclib plus letrozole improved OS for patients with <3 metastatic sites (HR, 0.78; 95% CI, 0.61–1.00) and for patients with ≥3 metastatic sites (HR, 0.71; 95% CI, 0.51–0.98). The survival benefit with ribociclib was similarly consistent regardless of prior adjuvant or neoadjuvant chemotherapy or endocrine therapy.

Overall findings from the MONALEESA biomarker and clinical subgroup analyses demonstrate consistent and long-term improvements in OS with the addition of ribociclib to ET in patients with HR+/HR– advanced breast cancer.^4,5^

## Notes

Please note that this content was created for the purpose of summarizing the content of the San Antonio Breast Cancer Symposium 2021 and has not been peer reviewed under the standard policies of *The Oncologist*.
